# Testing compliance to WHO guidelines for physical activity in Flanders insights from time-use diaries

**DOI:** 10.1186/s13690-019-0341-5

**Published:** 2019-03-20

**Authors:** Djiwo Weenas, Theun Pieter van Tienoven, Julie Verbeylen, Joeri Minnen, Ignace Glorieux

**Affiliations:** 1 Research group TOR, Department of Sociology, Vrije Universiteit Brussel, Pleinlaan 2, 1050 Brussels, Belgium; 2 Research group Interface Demography, Department of Sociology, Vrije Universiteit Brussel, Pleinlaan 2, 1050 Brussels, Belgium; 3 Social Policy Research Centre, University of New South Wales, Sydney, NSW 2052 Australia

**Keywords:** Physical activity, WHO guidelines, Time-diary, Time-use

## Abstract

**Background:**

Regular physical activity decreases the risk for numerous non-communicable diseases. The World Health Organization has suggested physical activity (PA) guidelines that, based on previous research, would provide health benefits to those who comply. The first guideline for health benefits suggests 150 min of moderate PA or equivalent per week. The guideline for *additional* health benefits suggests 300 min of PA or equivalent per week. The objective of this paper is to analyze to what extent these two WHO PA guidelines for adults are met in the Belgian region of Flanders. Furthermore, we are interested to see which groups are more or less likely to meet the PA guidelines.

**Methods:**

Crosstables and logistic regressions are used on a sample of 3028 adults in the Belgian region of Flanders. All respondents filled in a 7-day time-diary in which they continuously recorded all their activities.

**Results:**

Firstly, men are more likely than women to comply to both PA guidelines. Secondly, living with a partner increases the odds to comply to the guidelines. For men, this is the case for both guidelines, while for women, this only applies to the first guideline. Thirdly, women with a young child have lower odds to comply to the guidelines, while having a young child doesn’t have an effect for men.

**Conclusion:**

Previous research on meeting PA guidelines in Flanders shows diverging results. Time-diary data allows researchers to strictly follow the WHO definition when operationalizing compliance to PA guidelines. There is a need for future research that combines time-diaries with a PA questionnaire and accelerometer data to gain more insights on the benefits and pitfalls of both methodologies.

**Electronic supplementary material:**

The online version of this article (10.1186/s13690-019-0341-5) contains supplementary material, which is available to authorized users.

## Background

Time is essentially a democratic good, since everyone can spend 24 h a day, no more and no less. However, the way we spend our time can greatly influence our health outcomes. Physical inactivity has received acceptance as one of the prime risk factors for global mortality [1, 2]. Likewise, regular physical activity decreases risk of diabetes, coronary heart disease and stroke, hypertension, colon cancer, breast cancer and depression and more non-communicable diseases and health conditions [1–6].

In response to this awareness of the importance of physical activity, the World Health Organization (WHO) has issued global recommendations on physical activity for health [1]. The WHO recommends that adults aged 18–64 should do “*at least 150 minutes of moderate-intensity aerobic physical activity throughout the week, or do at least 75 minutes of vigorous-intensity aerobic physical activity throughout the week, or an equivalent combination of moderate- and vigorous-intensity activity”* (henceforth “Guideline of 150 min MPA”) to decrease the risk of non-communicable diseases and depression and to improve muscular and cardiopulmonary fitness [1].

A second WHO PA guideline for adults aged 18–64 states: *“For additional health benefits, […] should increase their moderate-intensity aerobic physical activity to 300 minutes per week, or engage in 150 minutes of vigorous-intensity aerobic physical activity per week, or an equivalent combination of moderate- and vigorous-intensity activity.”* (henceforth “Guideline of 300 min MPA”) [1].

The specific aim of this paper is to analyze, by means of time-use diaries, to which extent these two WHO PA guidelines are met in the Belgian region of Flanders. The use of time-diary data to study physical activity is relatively recent [7, 8], and this paper is the first to do so specifically in Belgium.

### Research on complying to WHO PA guidelines in Flanders

The results of previous research on compliance to the guideline of 150 min MPA in Belgium or Flanders are divergent.

Adilson et al. [9] tested compliance with the WHO guideline based on a single item of the 2012 European Social Survey: “On how many of the last 7 days did you walk quickly, do sports, or other physical activity for 30 min or longer”. They operationalized compliance to guideline of 150 min MPA by taking everyone into account that answered doing the above activities for at least 30 min for at least 5 days of the week. Their results for Belgium show that 68% of the women and 68.3% of the men comply to the guideline of 150 min MPA.

The Global status report on noncommunicable diseases 2014 from the WHO [10] also tests the guideline of 150 min MPA. It reports that 57.1% of the women and 67.5% of the men in Belgium comply to the guideline.

The European Commission and WHO regional office for Europe investigated PA in Belgium based on the 2013 Health interview Survey [11]. Vigorous PA was measured by asking “On how many of the last 7 days did you perform an activity requiring heavy physical exercise such as heavy lifting, digging, aerobic, jogging, football, …?” This was followed by the question: “On one of those days, how much time do you spend on heavy physical activity”. Similarly, moderate PA was measured by asking: “On how many of the last 7 days did you perform an activity requiring moderate physical exercise such as lifting a light load, cycling on a normal pace, or light sports?”, followed by the question on how many days of the week these activities took place. Compliance to PA guidelines was defined in this paper as engaging in moderate to vigorous physical activity for at least 30 min per day. The authors report that for Belgium, 48% of the men and 24% of the women comply to this guideline. For Flanders, 52% of the men and 28% of the women comply to this guideline.

The above shows that different methods are currently being used to test compliance to PA guidelines, yielding different results.

### Linking time-diary data with MET scores

Time-diary data provide information on all activities respondents have made over a certain period, usually ranging from one to seven days. Respondents provide rich and contextualized data by noting down the activities in which they are engaged, the location of the activity or if travelling, the mode of transport.

Tudor-Locke et al. were the first to link the Ainsworth Compendium of Physical Activities (henceforth ‘compendium’) with a time-use survey to study physical activity on a population level [8], and many have followed this rationale. The compendium lists 821 activities with their corresponding ‘metabolic expenditure of task’ (MET) scores as a measure of physical activity energy expenditure [12]. A MET value stands for the ratio of the work metabolic rate to the standard resting metabolic rate. Essentially, a MET score shows how physically demanding an activity is, compared to a situation at rest. A MET of 1 is defined as an oxygen uptake of 3.5 ml/kg/min (the oxygen cost of sitting quietly) or as 1 kcal/kg/h (the energy cost of sitting quietly) [12]. For example, based on the compendium, ‘ironing’ is assigned a score of 1.8 METs, and ‘vacuuming’ a score of 3.3 METs. This implies that ‘ironing’ and ‘vacuuming are 1.8 and 3 times more physically intensive than sitting quietly.

Combining this compendium with time-diary data thus creates a powerful tool to analyze physical activity, as we know for each respondent how physically active they were during their week, taking into account all activities. Since respondents filled in ‘work’ as a single category, we used the rationale as described in the paper of Deyaert et al. to assign working time PA [7].

### Influences on PA

Several sociodemographic factors are known to correlate with PA. First, men are more physically active than women, both in Europe and in the US [11, 13]. Second, energy expenditure differs between occupations [14], but the type of occupation also has an influence on other life domains such as the leisure-time physical activity [15, 16]. Third, both having a partner or living with a young child has an influence on physical (in)activity [17, 18]. Finally, health status also correlates with PA [19]. Apart from these sociodemographic factors, seasonality is also likely to have an effect on PA [20].

## Methods

### Data

Most publicly available time-use datasets, such as the American Time Use Survey or the Harmonised European Time use Survey are insufficient to test these WHO PA guidelines as those datasets consist of only one or two 24 h periods. The Flanders Modular Online Time Use Survey (MOTUS) data used in this paper consists of a 7-day diary. This 7-day scope lends itself well to see to what extent the WHO guidelines on physical activity are met, as those guidelines refer to a one week period. Although this longer timeframe makes data collection harder, it has the added benefit of capturing weekly routines. As a social fact, human behavior is organized in weekly cycles [21]. A 7-day time-use registration is more suited to measure moderate to vigorous physical activities that are performed as part of a weekly pattern rather than on a daily basis. For example, take a respondent that has a weekly routing of jogging once or twice a week. Using a one or two day diary, it is more likely that this activity is not captured. Therefore a one-week data collection is preferred when it comes to capturing weekly cycles.

Time-diary research is known to be demanding for respondents. The demanding procedure leads to rather low response rates. Therefore a large random population sample of 39,756 adults between the age of 18 and 75 were sent an invitation letter to participate in the research [21]. Data collection started in January 2013 and finished in February 2014. Obviously, due to seasonal changes in weather and due to social time constructs, the time of the year has an impact on the way people spend their time. To take this into account, a different subsample was asked to start with their time-use registration every other week, spread out over a whole year. 31.5% of the sample started participation in the research. Ninety percent of those who started also completed the pre-questionnaire of 30 min. But the time-diary registration remains a big hurdle for respondents: recording all activities during 7 days. After dropout and removing diaries with insufficient quality, 3260 respondents completed their time-diaries for the entire week. However, Table 1 shows that the non-response per stage does not seem to be very selective with regards to gender, age or education. More elaborate explanations on the data collection can be found in the paper of Minnen et al. [21].

**Table 1:** Response by population characteristics (in %)

	Population sample	Respondents	Respondents starting time-diary	Respondents completed at least one diary day	Respondents completing MOTUS
Gender					
Male	50.1	52.8	48.8	44.1	43.9
Female	49.9	47.2	51.2	55.9	56.1
Age					
18–24 yrs	10.9	13.2	13.9	13.7	13.3
25–39 yrs	25.2	26.1	26.7	26.2	24.7
40–54 yrs	30.2	31.3	30.3	31.6	32.0
55–64 yrs	18.3	18.8	18.1	19.5	20.1
65–75 yrs	15.3	10.7	11.0	9.0	9.8
Level of education^a^					
Low	29.5	22.3	25.5	15.3	14.7
Medium	39.3	35.7	36.9	34.6	34.0
High	31.2	42.0	37.5	50.1	51.3

^a^Distribution of level of education in weighted population sample based on Belgian Labour Force Survey 2012 (Flanders only), Source: National Register 2012 (population sample gender and age), Belgian Labour Force Survey 2012 (population sample education), MOTUS 2013 (respondents)

Table reprinted from Minnen et al. [21]

After selecting respondents aged 18–65, our dataset is comprised of 3028 respondents. This age group is specifically targeted by the WHO PA guidelines that are tested in this paper. Descriptive statistics of our variables are shown in Table 2.

**Table 2:** Descriptive statistics

	%	Count
Gender		
Male	42.4	1283
Female	57.6	1745
Age		
18–24 yrs	14.8	441
25–34 yrs	20.0	597
35–44 yrs	20.5	610
45–54 yrs	24.3	725
55–64 yrs	20.4	609
Occupation		
White-collar	45.2	1369
Unemployed	8.5	257
Blue-collar	8.9	269
Self-employed	14.1	427
Retired	9.6	290
Living with partner		
Yes	70.3	2130
No	29.7	898
Living with child < 7 yrs		
Yes	15.0	454
No	85.0	2574

### Indicators

All analyses are stratified by sex because we expect the mechanisms that explain physical activity to differ by gender.

The independent variables are occupation, age, living with a partner and living with a child under the age of 7.

Occupation was operationalized by asking respondents whether they work, and if that is the case, which category best matches their occupation. Afterwards, we recoded the answers to these two questions into the categories ‘unemployed’, ‘white-collar workers’, ‘blue-collar workers’, ‘self-employed’ and ‘retired’. Next, we took into account whether the respondents live with a partner and whether respondents live with a child younger than 7. Compliance with the WHO guidelines was calculated by strictly following the WHO definition. For each minute of moderate PA (MET of 3.0 to 5.9), the score of a respondent increased by 1, whilst for each minute of vigorous PA (MET of 6 or higher), the score of a respondent increased by 2. If this score for their week is 150 or higher, respondents comply to the guideline of 150 min MPA. Respondents comply to the guideline of 300 min MPA if this score for their week is 300 or higher.

### Analyses

For the analyses, a bivariate crosstable is used to investigate the relationship between gender and compliance with the WHO guidelines. Next, multivariate logistic regressions are applied to test compliance to the guidelines. The tables show the odds ratio (OR), 95% confidence interval and *p*-values. The multivariate logistic regressions are controlled for seasonality (month of the year) and self-reported health. All analyses were run in SPSS 25.

## Results

Results for meeting the guideline of 150 min MPA are shown in Table 3 and Table 4.

**Table 3:** Estimated compliance with WHO physical activity guidelines for health benefits in Flanders in 2013^a^

	Men		Women		Total	
	Percent	Count	Percent	Count	Percent	Count
No compliance WHO PA guideline	16.7%	214	20.1%	351	17.4%	565
Compliance with WHO PA guideline	83.3%	1069	79.9%	1394	82.6%	2463
Total	100.0%	1283	100.0%	1745	100.0%	3028

^a^Guideline of 150 min. Moderate PA or 75 min. Vigorous PA or equivalent

Fisher’s exact test of chi squared (2 sided) significance = 0.018

**Table 4:** Logistic regression on compliance to the WHO PA guideline for health benefits^a^

	Men				Women			
		95% CI				95% CI		
	OR	LL	UL	p	OR	LL	UL	p
Age				.009				.010
18–24 yrs. (ref)								
25–34 yrs	3.811	1.631	8.907	.002	1.340	.724	2.480	.351
35–44 yrs	3.361	1.425	7.928	.006	2.306	1.233	4.312	.009
45–54 yrs	3.024	1.295	7.063	.011	1.744	.942	3.228	.077
55–64 yrs	1.819	.753	4.394	.184	2.538	1.220	5.279	.013
Occupation				.000				.033
White-collar (ref)								
Unemployed	1.981	.727	5.398	.181	2.020	0.993	4.110	**.052**
Blue-collar	2.650	1.494	4.698	.001	1.680	.880	3.208	.116
Self-employed	1.662	.974	2.836	.063	.894	.621	1.287	.546
Retired	4.990	2.215	11.244	.000	2.876	1.030	8.030	.044
Living with partner	1.920	1.176	3.133	.009	1.594	1.120	2.269	.010
Living with child < 7 yrs	.591	.348	1.004	.052	.498	.333	.746	.001
Nagelkerke r^2	.142				.092			

^a^Guideline of 150 min moderate PA, 75 min vigorous PA, or equivalent

Controlled for self-reported health and month of the year

Table 3 shows that 82.6% complies to the guideline for health benefits. A slightly higher percentage of men (83.3%) than women (79.9%) complies to this guideline. Of course, this implies that 16.7% of the men and 20.1% of the women do not perform enough physical activity to meet the WHO guideline for health benefits.

Table 4 shows multivariate results on compliance with the guideline of 150 min MPA. Male blue-collar workers have 2.650 times higher odds to meet the guideline compared to male white-collar workers. A remarkable result is that retired men (OR 4.990) and women (OR 2.876) (not older than 65) are also more likely to comply to the guideline of 150 min MPA.

Living together with a partner leads to a higher likelihood of meeting the WHO guideline for health benefits, both for men (OR 1.920) and for women (OR 1.594).

The last and most striking result of Table 4 is that for men, there is no significant effect of living with a young child, whilst for woman, living with a young child lowers their odds of meeting the WHO guideline for health benefits by roughly 50% (OR 0.495).

Tables 5 and 6 show the result for the guideline of 300 min MPA. In Table 5, we see that 64.4% of the population in Flanders complies with this guideline for additional health benefits. For this guideline, the discrepancy between men and women is more apparent. 71.6% of the men comply to this guideline, compared to only 59.1% of the women.

**Table 5:** Estimated compliance with WHO physical activity guidelines for additional health benefits in Flanders in 2013^a^

	Men		Women		Total	
	Percent	Count	Percent	Count	Percent	Count
No compliance WHO PA guideline	28.4%	364	40.9%	714	35.6%	1078
Compliance with WHO PA guideline	71.6%	919	59.1%	1031	64.4%	1950
Total	100.0%	1283	100.0%	1745	100.0%	3028

^a^Guideline of 300 min. Moderate PA or 150 min. Vigorous PA or equivalent

Fisher’s exact test of chi squared (2 sided) significance < 0.001

**Table 6:** Logistic regression on compliance to the WHO PA guideline for *additional* health benefits^a^

	Men				Women			
		95% CI				95% CI		
	OR	LL	UL	p	OR	LL	UL	p
Age				.264				.000
18–24 yrs. (ref)								
25–34 yrs	1.820	.857	3.866	.119	2.232	1.250	3.986	.007
35–44 yrs	1.457	.683	3.105	.330	2.990	1.684	5.310	.000
45–54 yrs	2.063	.965	4.411	.062	3.860	2.173	6.858	.000
55–64 yrs	1.638	.739	3.631	.224	3.969	2.109	7.469	.000
Occupation				.000				.000
White-collar (ref)								
Unemployed	1.236	.553	2.763	.605	3.729	2.078	6.690	.000
Blue-collar	2.567	1.636	4.029	.000	1.981	1.211	3.242	.006
Self-employed	1.162	.778	1.736	.463	1.203	.891	1.624	.228
Retired	2.825	1.543	5.173	.001	2.194	1.200	4.013	.011
Living with partner	1.836	1.234	2.732	.003	1.166	.871	1.559	.302
Living with child < 7 yrs	.943	.617	1.441	.785	.604	.430	.849	.004
Nagelkerke r^2	.131				.126			

^a^Guideline of 300 min moderate PA, 150 min vigorous PA, or equivalent

Controlled for self-reported health and month of the year

Table 6 shows that for women, the older, the more likely they are to meet the guideline of 300 min MPA.

Both men and women are more likely to meet the guideline of 300 min MPA if they are unemployed, blue-collar worker or retired (not older than 65) compared to white-collar workers.

Having a partner gives men a 1.836 higher odds to meet the guideline of 300 min MPA. For women, having a partner does not have a significant effect on their likelihood of meeting the guideline for additional health benefits.

Lastly, living with a child under the age of 7 has no significant effect on meeting the guideline of 300 min MPA for men, whilst women who have a child under the age of 7 have much lower odds (OR 0.604) of meeting the WHO PA guideline for additional health benefits. Additional file 1 shows our extended tables, with the effects of our control variables month of the year and self-reported health.

## Discussion and limitations

Although there is a clear definition of PA guidelines from the WHO, previous research on meeting PA guidelines in Flanders and Belgium yielded different results. This is mainly because compliance to the WHO PA guidelines was operationalized in different ways (e.g.: [9–11]). The added value of time-diaries in this paper is that it allows us to strictly follow the WHO guideline in our operationalization.

Compared to previous research in the Belgian region of Flanders, the results of this paper show a higher compliance to the WHO PA guideline of 150 min of moderate PA or 75 min of vigorous PA or equivalent.

First, we believe this is at least partly because a lot of activities that contribute to moderate PA, such as vacuuming, are not reported when using a questionnaire on PA. A commonly used survey question is: “On how many of the last 7 days did you perform an activity requiring moderate physical exercise such as lifting a light load, cycling on a normal pace, or light sports?”. We assume it likely that a lot of activities that should fall under moderate PA, such as vacuum cleaning, are not considered when using a questionnaire. A time-use diary does not require the respondent to quickly make a summary of last week’s activities, categorizing all their activities according to the right PA intensity level and summing up the duration of all appropriate activities. Using time-diaries, respondents simply note down what activity they were doing.

Second, respondents are not biased due to a leading survey question. During time-diary data collection, there is no predefined research question as data could be used to analyze all sorts of topics, ranging from commuting, sleep time, childcare or working hours to PA research. The absence of priming respondents with specific PA questions lowers social desirability bias.

Furthermore, we believe time-diaries to be an improvement for PA research as it is less prone to recall bias [22].

However, there is still a need for future research that combines time-diaries with a PA questionnaire and accelerometer data in Flanders and elsewhere to gain more insights into the benefits and pitfalls of both methodologies.

In general, this research indicates that adult men are more likely to comply to the WHO guidelines than adult women. Even though the percentage that meets the WHO guidelines is high, it is important to keep in mind that this still implies that among adults in Flanders, 16.7% of the men and 20.1% of the women do not perform enough physical activity.

Furthermore, the effect of having a child on meeting the WHO PA guideline for additional health benefits differs between men and women. Previous research reported a decrease of PA when having dependent children, both for men and for women [18]. In our research, however, we found no significant effect on the likelihood of meeting any of the WHO PA guidelines for men with young children. For women, on the other hand, having a young child does significantly lower the likelihood of complying to both the WHO PA guidelines. These differences could be explained by the fact that women spend more time on childcare and household activities than men [23, 24]. This leaves them with less occasions, time or energy to spend on (vigorous) physical activities. Also, when fathers do partake in childcare, they are more likely to take up interactive and leisurely childcare such as playing with the children [25, 26]. Future interventions to promote PA could take into account that current gender roles play a part in the finding that living with a young child has a more negative impact on the likelihood of women to meet the WHO PA guidelines than for men.

When comparing occupations, whenever there was a significant effect between the reference category of white-collar workers with the other categories, it showed that white-collar workers were less likely to meet the PA guidelines. In accordance with previous research, this group continues to be an important focus for future interventions [14–16].

Contrary to previous research, this paper not only tests compliance to the WHO guideline for health benefits of 150 min of moderate PA or equivalent, but also to the WHO guideline for *additional* health benefits of 300 min of moderate PA or equivalent. Given the demonstrated additional health benefits, we suggest future research to test compliance to the guideline of 300 min MPA as well. [1].

It should be noted that the WHO guidelines on PA should not be the only focus for interventions and future research. Sedentary activity also associates with health outcomes, independent from PA levels [27, 28].

The response rate is a limitation of this research. It could be that non-participants of the data collection are less physically active than participants. However, the non-response is not very selective in terms of gender, age or education (Table 1), and result were controlled for self-reported health. Future research should try to overcome this non-response rate, for example by giving a reward to the participants or by staying more in touch with the respondents.

Even so, time-diary data fills a gap between questionnaires and accelerometer PA research. Questionnaires on the one hand are less valid, but can approximate PA with as little as 1 to 6 questions, without much effort and for a large sample. Accelerometer data on the other hand is considered the ‘gold standard’ for measuring PA, but is often used in small, experimental settings. Further developing the track of measuring PA with time-diary could prove an added value for public health research. Using time-diary data opens a lot of possibilities for PA research, because many countries already collect these data and make them publicly available. Some examples are the American Time Use Study and countries that participate in the MTUS dataset (Austria, Bulgaria, Canada, Finland, France, Hungary, Israel, Italy, Netherlands, Spain and the United Kingdom).

## Conclusion

The aim of this paper was to test compliance to two existing WHO guidelines for physical activity for adults aged 18 to 64 in the Belgian region of Flanders. Meeting the first guidelines for PA requires 150 min of moderate PA or equivalent, while meeting the second guideline for PA requires 300 min of moderate PA or equivalent. Compliance with these guidelines was tested by analyzing 7-day time-use diaries.

Our first main finding was that men are more likely to meet PA guidelines than women. 83.3% of the men and 79.9% of the women comply to the first guideline while 71.6% of the men and 59.1% of the women comply to the second guideline.

Secondly, having a partner was found to increase the odds of meeting PA guidelines, although for women this only applies to the first guideline.

Thirdly, having a young child decreases the likelihood of meeting the first guideline for both men and women. However, the likelihood of meeting the second guideline does not differ between men with and without a young child. Women with a young child are less likely than women without a young child to meet this second guideline.

Lastly, whenever a significant effect was found between white-collar workers and the other occupational categories (unemployed, blue-collar workers or self-employed), white collar-workers had lower odds of complying to the WHO guidelines. However, the effect of occupation was not consistent throughout the different models.

## Additional file

Additional file 1: Shows our an extended version of Table 4 and Table 6, including the effects of our control variables 'month of the year' and 'self-reported health'. (PDF 255 kb)

## Abbreviations

compendium: Compendium of physical activities
MET: Metabolic expenditure of task
MOTUS: Modular online time use survey
OR: Odds ratio
PA: Physical activity
WHO: World Health Organization
