# Supplementary material for: Testing compliance to WHO guidelines for physical activity in Flanders insights from time-use diaries
Source: Arch Public Health. 2019 Mar 20;77:16. doi: 10.1186/s13690-019-0341-5 (PMC6429766; doi:10.1186/s13690-019-0341-5)
Supplement: Supplementary file 1 — Shows our an extended version of Table 4 and Table 6, including the effects of our control variables 'month of the year' and 'self-reported health'. (PDF 255 kb) [file 13690_2019_341_MOESM1_ESM.pdf]

Table 4 (extended)
Logistic regression on compliance to the WHO PA guideline for health benefits [a], extended with control variables

	**Men**				**Women**			
		95% CI				95% CI		
	OR	LL	UL	p	OR	LL	UL	p
**Age**				.009				.010
18 - 24 yrs (ref)								
25 - 34 yrs	3.811	1.631	8.907	.002	1.340	.724	2.480	.351
35 - 44 yrs	3.361	1.425	7.928	.006	2.306	1.233	4.312	.009
45 - 54 yrs	3.024	1.295	7.063	.011	1.744	.942	3.228	.077
55 - 64 yrs	1.819	.753	4.394	.184	2.538	1.220	5.279	.013
**Occupation**				.000				.033
White-collar (ref)								
Unemployed	1.981	.727	5.398	.181	2.020	0.993	4.110	.052
Blue-collar	2.650	1.494	4.698	.001	1.680	.880	3.208	.116
Self-employed	1.662	.974	2.836	.063	.894	.621	1.287	.546
Retired	4.990	2.215	11.244	.000	2.876	1.030	8.030	.044
**Living with partner**	1.920	1.176	3.133	.009	1.594	1.120	2.269	.010
**Living with child <7 yrs**	.591	.348	1.004	.052	.498	.333	.746	.001
**Month of the year**				.018				.407
December (ref)								
January	.834	.388	1.793	.641	.624	.330	1.180	.146
February	.924	.461	1.849	.823	.691	.382	1.249	.221
March	2.338	.856	6.383	.097	1.028	.478	2.211	.944
April	2.334	1.046	5.207	.038	1.192	.622	2.283	.597
May	2.979	1.164	7.621	.023	.740	.372	1.471	.391
June	1.875	.776	4.530	.162	1.628	.737	3.599	.228
July	5.778	1.259	26.522	.024	1.287	.516	3.210	.588
August	3.126	1.086	9.002	.035	.850	.423	1.708	.648
September	1.439	.678	3.055	.343	.820	.442	1.523	.531
October	1.371	.677	2.778	.381	.679	.369	1.249	.214
November	1.057	.513	2.179	.881	.782	.403	1.516	.466
**Self-reported good health**	3.659	2.071	6.464	.000	1.671	1.066	2.619	.025
Nagelkerke r^2		.142				.092		

[a] Guideline of 150 min moderate PA, 75 min vigorous PA, or equivalent

Table 6 (extended)
Logistic regression on compliance to the WHO PA guideline for health benefits [a], extended with control variables

	Men				Women			
		95% CI				95% CI		
	OR	LL	UL	p	OR	LL	UL	p
**Age**				.264				.000
18 - 24 yrs (ref)								
25 - 34 yrs	1.820	.857	3.866	.119	2.232	1.250	3.986	.007
35 - 44 yrs	1.457	.683	3.105	.330	2.990	1.684	5.310	.000
45 - 54 yrs	2.063	.965	4.411	.062	3.860	2.173	6.858	.000
55 - 64 yrs	1.638	.739	3.631	.224	3.969	2.109	7.469	.000
**Occupation**				.000				.000
White-collar (ref)								
Unemployed	1.236	.553	2.763	.605	3.729	2.078	6.690	.000
Blue-collar	2.567	1.636	4.029	.000	1.981	1.211	3.242	.006
Self-employed	1.162	.778	1.736	.463	1.203	.891	1.624	.228
Retired	2.825	1.543	5.173	.001	2.194	1.200	4.013	.011
**Living with partner**	1.836	1.234	2.732	.003	1.166	.871	1.559	.302
**Living with child <7 yrs**	.943	.617	1.441	.785	.604	.430	.849	.004
**Month of the year**				.000				.007
December (ref)								
January	1.069	.558	2.048	.842	.878	.525	1.466	.618
February	.969	.544	1.727	.916	1.006	.628	1.611	.980
March	3.031	1.344	6.835	.008	1.069	.597	1.914	.823
April	3.212	1.660	6.218	.001	1.909	1.155	3.152	.012
May	2.609	1.303	5.224	.007	1.067	.619	1.841	.815
June	2.352	1.144	4.839	.020	2.432	1.356	4.361	.003
July	3.190	1.263	8.056	.014	1.195	.623	2.291	.592
August	1.935	.938	3.995	.074	.893	.514	1.553	.689
September	2.049	1.084	3.874	.027	1.046	.644	1.698	.855
October	1.273	.719	2.256	.408	1.004	.611	1.650	.988
November	1.125	.620	2.042	.698	.721	.429	1.214	.219
**Self-reported good health**	3.081	1.824	5.206	.000	1.627	1.093	2.423	.017
Nagelkerke r^2		.131				.126		

[a] Guideline of 300 min moderate PA, 150 min vigorous PA, or equivalent
